# *Drosophila* as a model for homeostatic, antibacterial, and antiviral mechanisms in the gut

**DOI:** 10.1371/journal.ppat.1006277

**Published:** 2017-05-04

**Authors:** Xi Liu, Jeffrey J. Hodgson, Nicolas Buchon

**Affiliations:** 1 Cornell Institute for Host Microbe Interactions and Disease, Department of Entomology, Cornell University, Ithaca, New York, United States of America; 2 Boyce Thompson Institute, Cornell University, Ithaca, New York, United States of America; Nanyang Technological University, SINGAPORE

## A conserved midgut structure from fly to human

**Fig 1:**
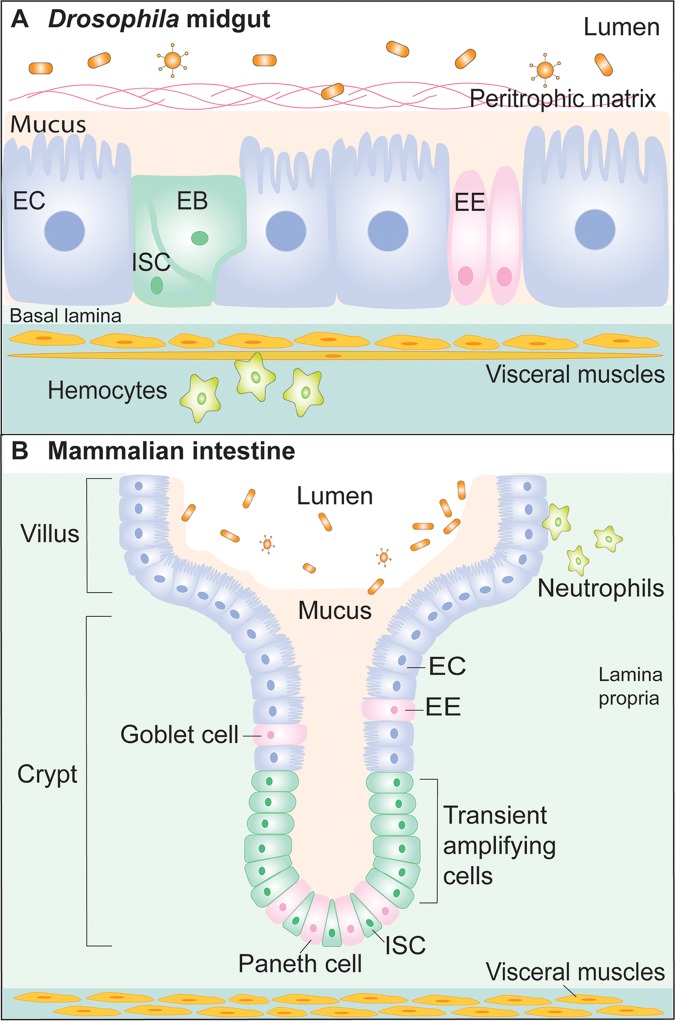
Parallels between the *Drosophila* and mammalian gut epithelia. (**A**) The fly midgut is composed of absorptive enterocytes (ECs) and secretory enteroendocrine cells (EEs) that arise from differentiation of the basally embedded intestinal stem cells (ISCs). Enteroblasts (EBs) are transient progenitors destined to differentiate into ECs. The epithelium is protected by the peritrophic matrix and thin mucus layer apically and is sheathed in a basal lamina and visceral muscle cells. (**B**) Similarly, the mammalian intestinal epithelium is composed of progenitor and Paneth cells residing at the base of crypts and absorptive cells (ECs) and secretory cells (EE and Goblet cells) that progress towards the apex of the villus. The mucus layer protects the gut epithelial cells from direct contact with commensal microbes. Hemocytes (**A**) or Neutrophils (**B**) transmit secreted signals to the gastrointestinal tract.

The gastrointestinal (GI) tract serves as an active barrier and a first layer of defense against the numerous microbes that populate the gut lumen. The fly GI tract is composed of self-renewing digestive and absorptive tissues and shares several properties with the mammalian counterparts, the stomach, small intestine, and colon. The gut epithelium is physically protected by the mucus layer in mammals and by a chitinous peritrophic matrix (PM) in *Drosophila* [1] (Fig 1). Underneath the protective layer is an epithelial monolayer surrounded by a basal lamina and visceral muscles (Fig 1). In both *Drosophila* and mammals, gut tissue maintenance is extremely important to help maintain physical barrier integrity and proper immune function. The GI epithelium is continuously renewed by intestinal stem cells (ISCs). In flies, ISCs self-renew and give rise to either a transient enteroblast (EB) that terminally differentiates into an absorptive enterocyte (EC) or a pre-enteroendocrine cell that becomes a secretory enteroendocrine cell (EE) [2] (Fig 1A). Similarly, in mammals, ISCs self-renew and differentiate into intermediate cell types the transit amplifying cells, which proliferate and further differentiate into ECs or secretory cells (EEs, Goblet cells, and Tuft cells), and dedicated Paneth cell progenitors that mature into Paneth cells (Fig 1B). This striking structural similarity, the fact that several key signaling pathways involved in immunity and tissue regeneration are conserved from *Drosophila* to humans, and the development of cutting edge techniques, including live imaging and RNA-seq of select cell types in the midgut [3], make the *Drosophila* midgut an ideal model for revelatory studies of host–microbiome associations, innate immunity, tissue regeneration, and arbovirus–vector interactions.

## *Drosophila* as a model to dissect host interaction with its gut microbes

The microbial diversity in the *Drosophila* gut is lower compared to that of mammals. A major difference is that the *Drosophila* gut lumen is likely more of an aerobic environment because of its limited size, in contrast to some parts of the mammalian GI tract. Although around 30 bacterial species have been identified in the midgut of *Drosophila*, *Acetobacter* and *Lactobacillus* are the two genera predominantly isolated from both wild-caught and laboratory-reared flies [4–9]. Germ-free and derivative gnotobiotic flies (i.e., reassociated with one or more bacteria) provide a less complex approach for in-depth analyses of the impact individual microbes have on gut and/or whole fly homeostasis. For example, *Acetobacter pomorum* and *Lactobacillus plantarum* trigger the insulin and Target of Rapamycin (TOR) pathways (respectively), both of which provide growth advantages to fly larvae in limiting nutrient conditions [10,11]. Similarly, *L*. *plantarum* was found to benefit the growth of infant mice during chronic undernutrition [12]. Studies in flies have shown that the gut microbiota can also become deleterious with age. In aged flies, the load and diversity of gut microbes increase, perhaps as a consequence of immune dysregulation, and this dysbiosis impairs gut function, ultimately driving mortality [13–16].

The gut microbiota has significant effects on midgut immune responses and epithelium physiology [17]. Gut microbes increase the tightly regulated basal level of NF-κB pathway-dependent immune activity, and NF-κB–induced antimicrobial peptides limit bacterial growth in a feedback loop [13,18–22]. In addition, certain bacteria, including *Lactobacilli*, trigger production of reactive oxygen species (ROS) by the NADPH oxidases Duox and Nox [23–25]. In *Drosophila*, ROS (1) are directly antimicrobial, (2) promote secretion of cytokines that result in para- and autocrine JAK-STAT and/or Jun N-terminal kinase (JNK) signaling [13], and (3) synergize with NF-κB–mediated responses to control microbial invaders [26]. *Drosophila* gut microbes also affect the cellular composition of gut epithelium [6]. Likewise, the mammalian gut microbiota has also been proposed to influence intestinal development and function [27], possibly by promoting cytokine signaling and reparative inflammation [28]. Therefore, elucidating the dialogue between *Drosophila* and its gut microbiota will benefit studies on gut development, homeostasis, and physiology across otherwise disparate animals.

## The gut response to pathogens: From mucosal immunity to tissue repair

Bacterial pathogens are also controlled by the conjunction of physical barriers and the production of ROS and antimicrobial peptides, but the immune responses are induced to a higher level compared to that caused by the microbiota [6,29]. In mammals, intracellular intestinal pathogens such as *Salmonella*, *Listeria*, and *Shigella* are commonly used, while in *Drosophila*, most pathogens studied are extracellular gram-negative bacteria (e.g., *Pectinobacteria*, *Pseudomonas*, and *Serratia*). In mice, intestinal infections trigger NF-κB activation downstream of Toll-like receptors (TLRs) and Nod-like receptors (NLRs). Similarly, activation of the Immune Deficiency (Imd) pathway in *Drosophila* depends on both membrane-bound (PGRP-LC) and cytoplasmic (PGRP-LE) receptors [30,31]. In mice, the intestine relies on the lumenal secretion of antimicrobial peptides by Paneth cells as well as the recruitment of immune cells such as neutrophils to prevent infection [32]. Recently, a role for hemocytes, the circulating immune cells of *Drosophila*, has been described in controlling inflammatory signaling and intestinal regeneration in the gut [33–35], suggesting that the interplay between immune cells and the gut epithelium is also conserved from flies to mammals.

**Fig 2:**
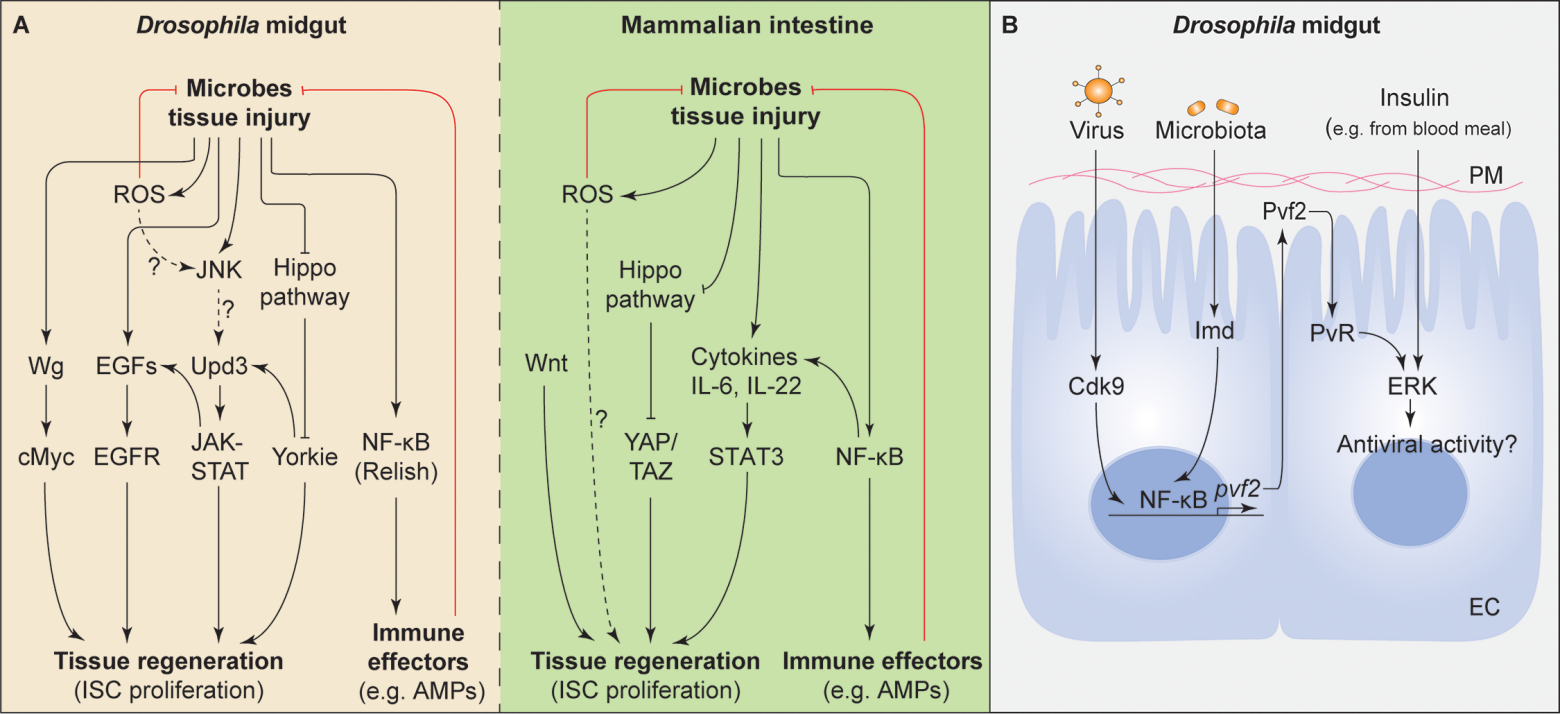
A conserved gene regulatory network controls tissue homeostasis in flies and mammals. (**A**) In both flies and mammals, the gut epithelium produces immune effectors, including reactive oxygen species (ROS) and antimicrobial peptides (AMPs). Epithelial cells and immune cells secrete cytokines that stimulate tissue regeneration. The Hippo pathway is a conserved regulator of intestinal stem cell (ISC) activity. In *Drosophila*, activation of the JAK-STAT pathway by the cytokine Unpaired 3 (Upd3) triggers the release of epidermal growth factors (EGFs) by the stem cell niche, which then induces stem cell proliferation. JAK-STAT activation also directly stimulates ISC proliferation and differentiation. The Wingless (Wnt/Wg) pathway is a major regulator of ISC proliferation in mammals and also promotes tissue regeneration through cMyc in the infected *Drosophila* midgut. The dashed arrows indicate presumed activities but as yet are undefined. (**B**) Gut microbes and viruses coordinately stimulate a PDGF-VEGF Receptor and Extracellular Signal-Regulated Kinase dependent (pvf2/PVR/ERK) antiviral response through the Immune Deficiency (Imd) pathway and Cyclin-dependent-kinase 9 (Cdk9) induction, respectively, in the midgut of *Drosophila*. The ERK-stimulated antiviral activities/effectors have not been determined. In addition, exogenous insulin initiates ERK mediated antiviral activity.

Tissue repair and regeneration is integral to maintaining intestinal homeostasis in both healthy and disease states. GI recovery from microbial pathogenesis relies largely on cellular replenishment by the ISCs. As a consequence, pathogenic infection-induced ISC proliferation synergizes with oncogenic lesions to promote tumor formation [36]. Evolutionarily conserved pathways such as JAK-STAT, epidermal growth factor receptor (EGFR), Wingless (Wg)/Wnt, and Hippo maintain ISC homeostasis in both insect and mammalian models [28,29]. Unpaired 3 (Upd3), a *Drosophila* member of the interleukin 6 (IL-6) family of cytokines, triggers JAK-STAT in progenitor cells (ISC and EB) and visceral muscles [13,37,38]. JAK-STAT activation in progenitor cells stimulates ISC proliferation and EB differentiation (Fig 2A). In addition, JAK-STAT signaling also reprograms the stem cell niche to secrete epidermal growth factors (EGFs), thus indirectly promoting ISC proliferation [39–41]. IL-6 and IL-22 have been shown to mediate inflammation regulated tissue regeneration upon GI injury in mice [42,43] (Fig 2A). Hippo signaling also regulates ISC proliferation in homeostasis and upon stress [44,45] (Fig 2A). Wg/Wnt signals in both models are essential to maintaining ISC activity for tissue homeostasis (Fig 2A). Upon damage to fly guts, Wg secreted by ISCs and EBs elicits cMyc-dependent ISC proliferation (Fig 2A) [46]. In *Drosophila*, diverse mitotic signals converge on regulating calcium (Ca^2+^) oscillations in ISCs, and high cytoplasmic Ca^2+^ levels ultimately trigger ISC proliferation [47]. The conservation between fly and murine signaling pathways suggests that the *Drosophila* midgut will be an instrumental model for defining or clarifying how regenerative pathways are activated in response to microbes. It remains uncertain whether the homeostatic responses of the GI tract to pathogenic and indigenous microbes are the same, but future studies dissecting immune and damage responses induced by different microbes will delineate this.

## The *Drosophila* gut as a model for arbovirus–vector interactions

The emergence of several arboviruses impacting human health (e.g., Zika, dengue, chikungunya, Rift Valley fever virus) in the past decade has instigated vast research into arbovirus–vector interactions. Mosquitoes and other biting insects are natural virus vectors, but arboviruses belonging to the Flavivirus, Alphavirus, and Bunyavirus families can infect *Drosophila* experimentally, thus establishing a pertinent model to study innate immune signaling and other aspects of virus vectoring capacity [48]. Systemic virus infections of *Drosophila* and other insects are controlled by a combination of RNA interference (RNAi), apoptosis, and immune responses downstream of key signaling pathways (e.g., Toll, NF-κB, JAK-STAT) [49]. However, little is known about the activation and function of antiviral pathways in the insect midgut.

Most insect-specific viruses are sublethal upon oral infection but often lethal when injected into the hemocoel [49]. Viruses acquired orally by *Drosophila* adults [50,51] and larvae [52] face antiviral processes, which limit virus ability to breach the midgut barrier and spread systemically. The Toll pathway in *Drosophila* has recently been shown to limit viral infection initiated in the midgut. The Spätzle (Spz)-Toll-Dorsal pathway was required and sufficient to survive oral infection with Drosophila C virus (DCV) but, consistent with another study [53], this pathway did not have a role when the virus was injected directly into the hemocoel [50]. The realization in *Drosophila* that the nutrient-sensitive Extracellular signal-Regulated Kinase (ERK) pathway restricts infection by diverse orally acquired viruses has demonstrated a link between the nutritional status of the host and the function of the gut as an active barrier against infection [51]. Indeed, Xu et al. showed that ingestion of human insulin by *Drosophila* is capable of stimulating antiviral ERK activity in the midgut (Fig 2B) [51]. This antiviral ERK activity is also conserved in mosquito (*Aedes*) cells, suggesting that exposure to insulin during a blood meal could reduce the ability of arboviruses to naturally infect mosquitoes [51].

In insects, endosymbionts such as *Wolbachia* are potent regulators of viral infection and are used to control the vector capacity of some mosquitoes [54–56]. In mammals, the gut microbiota influences viral infectivity both positively and negatively, albeit via largely unknown mechanisms [57]. Recently, Sansone et al. have shown that midgut antiviral activity is primed by commensal microbes in the *Drosophila* midgut [58]. This study showed that the integration of two distinct microbial signals in the gut is necessary to stimulate antiviral ERK activity. First, peptidoglycan from the commensal bacterium *Acetobacter pomorum* activates the NF-κB cascade, leading to the transcriptional induction of the Platelet-Derived-Growth Factor/Vascular Endothelial Growth Factor (PDGF-VEGF) homologue, Pvf2 (Fig 2B). In parallel, oral exposure to several viruses (Sindbis, Vesicular Stomatitis Virus [VSV], Dengue virus [DENV-2], and Drosophila C Virus, [DCV]) triggers expression of the transcriptionally paused Cyclin-dependent-kinase 9 (Cdk9) that potentiates Pvf2 secretion (Fig 2B) and binding to the PDGF-VEGF Receptor (PVR) that in turn stimulates antiviral ERK signaling. Many other such details of insect GI antiviral mechanisms and virus–vector interactions are yet to be discovered using *Drosophila* in this era of (re)emerging arboviruses.

## Conclusions and future directions

The gut is a major interface between a host and microbes. Whether we consider the emerging notion that the gut microbiota influences host physiology, the relation of GI inflammation to health and disease, or that the gut of insect vectors is a first point of contact with human parasites, it is increasingly important to elucidate the complexity and plethora of interactions between the GI tract and microbes. Due to the strong conservation of both structure and function of the gut epithelium, the less complex microbial community that composes the *Drosophila* gut microbiota, and the ease of genetically manipulating *Drosophila*, the fruit fly will continue to thrive as a workhorse for biological discoveries in this area.
